# Iodine deficiency in pregnant women in Sweden: a national cross-sectional study

**DOI:** 10.1007/s00394-019-02102-5

**Published:** 2019-10-15

**Authors:** Sofia Manousou, Maria Andersson, Robert Eggertsen, Sandra Hunziker, Lena Hulthén, Helena Filipsson Nyström

**Affiliations:** 1 Institute of Medicine, Sahlgrenska Academy, University of Gothenburg, Göteborg, Sweden; 2 Frölunda Specialist Hospital, Marconigatan 31, 42144 Västra Frölunda, Sweden; 3 Division of Gastroenterology and Nutrition, University Children’s Hospital Zurich, Eleonore Foundation, Steinwiesstrasse 75, 8032 Zurich, Switzerland; 4 Iodine Global Network, Ottawa, ON Canada; 5 Mölnlycke Health Care Centre, Ekdalavägen 2, 43530 Mölnlycke, Sweden; 6 Human Nutrition Laboratory, Institute of Food Nutrition and Health, ETH Zurich, Schmelzbergstrasse 7, 8092 Zurich, Switzerland; 7 Department of Internal Medicine and Clinical Nutrition, Sahlgrenska Academy, University of Gothenburg, Medicinaregatan 13, 40530 Gothenburg, Sweden; 8 Department of Endocrinology, Sahlgrenska University Hospital, Gröna Stråket 8, 41345 Gothenburg, Sweden

**Keywords:** Iodine, Pregnancy, Sweden, Thyroglobulin, Urine iodine concentration, Iodine deficiency

## Abstract

**Purpose:**

Voluntary salt iodization at 50 mg/kg salt ensures adequate iodine nutrition in Swedish school-aged children, but iodine status in pregnant women is uncertain.

**Methods:**

We conducted a cross-sectional national study of 743 pregnant women, at median gestational age of 23 weeks (IQR 9, 38), recruited from maternal health care centers. We measured: urinary iodine concentration (UIC) and urinary creatinine concentration in spot urine samples; thyroglobulin (Tg), thyroid-stimulating hormone (TSH), and total thyroxine (tT4) on dried blood spots (DBS); and thyreoperoxidase antibodies in serum samples. Data on dietary supplement use were obtained, and women were classified as supplement users (consuming multivitamins containing ≥ 150 µg iodine/day) and non-supplement users (no supplements or < 150 µg iodine/day from supplements).

**Results:**

Overall median UIC [bootstrapped 95% confidence interval (CI)] was 101 µg/L (95, 108; *n *= 737): 149 µg/L (132, 164) in supplement users (*n *= 253) and 85 µg/L (79, 92) in non-supplement users (*n *= 440) (*p *< 0.001). Overall geometric mean DBS-Tg (95% CI) was 22.1 μg/L (20.8, 23.5; *n *= 675) and the prevalence of elevated DBS-Tg was 19%. DBS-Tg was lower in supplement users (*n *= 229) than in non-supplement users (*n *= 405) (19.1 vs 24.4 μg/L, *p *< 0.001). DBS-TSH, DBS-tT4, and S-TPOab positivity did not differ between the two groups.

**Conclusions:**

Pregnant women in Sweden have inadequate iodine nutrition. Women not taking iodine supplements containing ≥ 150 µg iodine/day are affected by mild iodine deficiency and are at higher risk for increased thyroid activity, while maintaining euthyroidism. Iodine intake should be improved in women both before and after conception by promotion of iodized salt instead of non-iodized salt. We urge regular monitoring of iodine status in the general Swedish population, as well as in risk groups.

## Introduction

Adequate dietary iodine is essential for the production of thyroid hormones. Moderate-to-severe iodine deficiency during pregnancy increases the risk of cognitive impairment in the offspring [1–4]. Observational studies suggest that even mild iodine deficiency during pregnancy may negatively affect verbal intelligence quotient and educational level in their children [5–7], although a causal relation could not be confirmed in a recent randomized controlled trial of iodine supplementation in pregnant women with mild iodine deficiency [8].

Pregnant women have higher iodine requirements than before pregnancy and are vulnerable to iodine deficiency due to increased thyroid hormone production, increased renal iodine clearance, and transplacental transfer of iodine to the fetus [9]. The recommended daily iodine intake for pregnant women is 250 μg, higher than the 150 μg/day recommended to non-pregnant women of reproductive age [10]. A median spot urinary iodine concentration (UIC) during pregnancy of 150–249 µg/L indicates adequate iodine nutrition in a population [10].

Iodine deficiency was historically endemic in Sweden with reported goiter prevalence up to 60% in certain areas [11, 12]. Swedish authorities introduced voluntary iodine fortification of table salt in 1936 [11, 13]. The current level of iodine in fortified salt is 50 mg/kg salt and has remained the same since 1966 [14, 15]. The first national monitoring of the present salt iodization program was conducted in school-aged children (6–12 years) in 2007 and the findings implied iodine sufficiency [16, 17]: no goiter was observed [16] and the median UIC was 125 µg/L [17], within the recommended range of 100–299 µg/L [10, 18]. At the same time, a local study suggested mild iodine deficiency in the third trimester of pregnancy [19]. Furthermore, more than 75% of consumed salt in Sweden is non-iodized [personal communication with the salt industry, 2017], milk iodine concentration has declined [20], consumption of iodine-rich fish and dairy products is decreasing [14], and national authorities recommend reduced salt intake [21].

We conducted a national cross-sectional study in Sweden with the objectives of assessing iodine status and thyroid function in pregnant women and evaluating the impact of prenatal iodine supplementation on iodine status and thyroid function.

## Materials and methods

### Study design

The sample collection period of this national cross-sectional study in pregnant women was August 2015 to April 2016. Subjects were recruited using a stratified two-stage probability proportionate to size cluster design [10]. Stratified random sampling was applied using the common grouping of the Swedish population into six regions (H regions) based on regional population density [22]. In the first stage, 25 maternal health care (MHC) centers were recruited. The number of selected centers corresponded to the total population of each stratum. If a selected MHC center declined participation, an alternate MHC center was randomly selected with replacement sampling from the same stratum. From each MHC center, midwives recruited 30 consecutive women, 10 from each trimester. We defined the first, second, and third trimesters as pregnancy weeks 1–12, 13–28, and ≥ 29, respectively.

Gothenburg Ethical Committee approved the study (Dnr 095-15 and T666-15) and it was performed in compliance with the ethical standards laid down in the 1964 Declaration of Helsinki and its later amendments. All study participants gave their informed consent prior to their inclusion in the study.

Each woman provided a spot urine sample for assessment of UIC and urinary creatine concentration (U-creatinine) and a dried blood spot (DBS) sample for measurement of thyroglobulin (Tg), thyroid-stimulating hormone (TSH), and total thyroxine (tT4). A venous blood sample was obtained for the determination of serum thyreoperoxidase antibodies (S-TPOabs) in the MHC centers with capacity to handle and store serum samples (13 of 25 centers).

The midwives obtained information on gestational age, presence of multiple pregnancy, and measured weight and height using standard anthropometric techniques. A brief questionnaire, filled in by the study participants, was used to collect information on current smoking, use of vitamin supplements, thyroid disease, and thyroid-related medication.

### Subjects

All pregnant women attending one of the selected MHCs were eligible for the study; no exclusion criteria were applied.

## Methods

Women were given a plastic cup and were asked to provide ~ 20 mL of fresh midstream urine, directly after their routine visit to MHC. Midwives were instructed not to expose the urine samples to any dipsticks, due to risk of iodine contamination. Samples were transported in a cool box from the place of collection to the central laboratory. Aliquots of all urine samples were frozen at − 20 °C until analysis. Blood drops (50 μL) were collected by a finger prick and directly collected onto filter paper cards (IDBS-226, Perkin Elmer, CT). The DBS cards were dried at room temperature, placed in sealed plastic bags, and stored at 4 °C until analysis or frozen at − 20 °C before analysis.

### Laboratory analyses

#### Urinary iodine and creatinine concentration

Spot urine samples were transported and stored at − 20 °C until analysis. For transport shorter than 8 h, they were transported in room temperature, which does not influence their quality. UIC in spot urine samples was measured by a single laboratory technician at the Department of Clinical Nutrition at Sahlgrenska Academy, University of Gothenburg (Göteborg, Sweden) using the Pino modification of the Sandell-Kolthoff reaction [23]. The laboratory successfully participates in the EQUIP network (US Centers for Disease Control and Prevention, Atlanta, GA) and is evaluated for analytical accuracy every 3 months. All urine samples were measured in duplicate and reanalyzed, if difference in absorbance was > 5% for UIC > 150 μg/L, > 10% for UIC 50–150 μg/L, and > 15% for UIC < 50 μg/L. External quality control was ensured by measuring control urine samples that were added to the daily sample measurements. The UIC analysis was validated against inductively coupled plasma mass spectrometry at the Genomics and Biomarkers Unit in Finland, as part of the EUthyroid project (Helsinki, Finland). The correlation between the two methods was high (linear regression: *R*^2^ = 0.875, *p *= 0.023). Adequate iodine status in pregnant women was defined as median UIC ≥ 150 µg/L [10].

U-creatinine was measured with a photometric method using Cobas from Roche Diagnostics (Stockholm, Sweden) at the accredited Laboratory of Clinical Chemistry at Sahlgrenska University Hospital, Göteborg, Sweden. The coefficience of variance was 4%.

#### Thyroglobulin concentration

DBS-serum samples were transported and stored at − 20 °C until analysis. For transport shorter than 8 h, they were transported in room temperature, which does not influence their quality. DBS-Tg was measured with a DBS-Tg sandwich enzyme-linked immunosorbent assay at ETH Zurich (Zurich, Switzerland) [24]. Serum control samples (Liquicheck Tumor Marker Control, LOT.19970 and LOT.19980; Bio-Rad, Hercules, CA, USA) were used as standards for the DBS-Tg assay. In-house DBS samples were used for quality control and the coefficient of variance (CV) was 14% (*n *= 21) at 19 µg/L and 9% (*n *= 21) at 67 µg/L. The assay-specific reference range for DBS-Tg in pregnant women was 0.3–43.5 µg/L [25].

#### TSH and tT4 concentrations

DBS-serum samples for TSH and Tt4 were handled at the same way as DBS-serum samples for Tg, described above. TSH and tT4 were measured in DBS samples at the Swiss Newborn Screening Laboratory, University Children’s Hospital (Zurich, Switzerland). DBS-TSH and DBS-tT4 were analyzed with the use of an automated time-resolved fluoroimmunoassay method (GSP 2021-0010; PerkinElmer Life Sciences, Turku, Finland) using a related GSP Neonatal TSH/tT4 kit (PerkinElmer Life Sciences, Turku, Finland). Kit-specific DBS controls were used for the analysis. The CV for TSH was 10% (*n *= 12) at 15 mU/L and 8% (*n *= 11) at 62 mU/L. For tT4, the CV was 9% (*n *= 12) at 40 nmol/L, 10% (*n *= 12) at 100 nmol/L, and 8% (*n *= 12) at 158 nmol/L.

#### Serum *thyreoperoxidase* antibodies

Serum samples were transported in − 20 °C and were thereafter stored at − 80 °C, until analys. S-TPOabs were measured in serum samples at the Laboratory for Clinical Chemistry at Skåne University Hospital (Malmö, Sweden) with an electrochemoluminicence immunoassay (Cobas NPU20041, Roche Diagnostics, Solna, Sweden). The Laboratory of Clinical Chemistry at Skåne University Hospital, Sweden, successfully participates in national, international and internal control programs, where unknown samples are analyzed, a process reviewed by Swedac, the Health and Social Care Inspectorate and by the Swedish Technician Research Institute. The normal reference range for S-TPOabs was < 34 kIE/L.

#### Thyroid morbidity

Kit- and laboratory-specific reference ranges were used to calculate the prevalence of thyroid dysfunction. We used trimester- and pregnancy-week-specific reference ranges. We used the normal reference values defined for DBS-TSH in non-pregnant adults (0.1–3.7 mIU/L) for pregnant women in the second and third trimesters and reduced the upper limit to 3.0 mIU/L in the first trimester, as recommended by the American Thyroid Association [26]. For DBS-tT4 in pregnant women in week 1–6, we applied the assay-specific normal reference range for non-pregnant adults of 65–165 nmol/L. Thereafter, we increased the non-pregnant upper reference limit by 5% per week, beginning with week 7 [26]. Starting week 16, we multiplied the non-pregnant adult reference range by 1.5 and used the resulting range of 97.5–247.5 nmol/L [29]. Low or high DBS-TSH represented hyper- and hypothyroidism, respectively, and this was described as clinical or subclinical thyroid disease depending on whether DBS-tT4 was outside or within the reference range, respectively. Normal DBS-TSH with high or low DBS-tT4 was defined as isolated hyper- or hypothyroxinemia, respectively.

### Statistical methods

The sample size of the study was determined to assess the median UIC with 5% precision (95% CI) [27–29]. The recommended sample size needed to accurately estimate the median (or geometric mean) UIC with 5% precision range from 389 to 473, based on an inter-individual variability (CV %) of 50–55% for transformed UIC data [27, 28]. A previous study in Swedish pregnant women [19] reported an inter-individual variability (CV %) for UIC of 17% for log data (71% for crude data), lower than estimated in the studies by König et al. and Karmisholdt et al. [27, 28]. However, to account for an assumed skewed population distribution of UIC and the cluster design, we aimed to enroll 750 pregnant women.We used Excel 2016 (Microsoft, Redmond, WA, USA) and SPSS version 24.0 (IBM, Armonk, NY, USA) for data processing and analysis. The primary outcome parameter of the study was UIC. Secondary outcome parameters were DBS-Tg, DBS-TSH, DBS-tT4, and S-TPOabs: subjects undergoing treatment with levothyroxine were excluded from the data analysis of these four outcomes.

Normality for continuous variables was assessed visually (by comparing with normal distribution, Q–Q plots, and box-plots) and by normality tests (Shapiro–Wilk test). Non-normally distributed data were log-transformed before data analysis. DBS-Tg fulfilled the normality criteria after log-transformation and is presented as geometrical mean and 95% confidence interval (CI). Variables remaining skewed after log-transformation are presented as median, quartile (Q)1, and Q3. Non-parametric 95% CIs around the median were obtained using the bootstrap technique (*n *= 1000). UIC data were normally distributed after log-transformation, but is presented as median, interquartile range (IQR), and bootstrapped 95% CI for consistency with common practice. No outliers were removed from the descriptive data.

Women were categorized as supplement users (using supplements containing ≥ 150 µg iodine/day) and non-supplement users (using no supplements or supplements containing < 150 µg iodine/day). The cut-off of 150 μg was chosen as this is the recommended level for iodine supplementation during pregnancy in many countries [26, 43] and as the most popular multivitamin tablets in Sweden contain 75, 80,100 and 150 μg iodine. Independent *t* test and ANOVA with Bonferroni correction were used for group comparison of normally distributed variables (after logarithmic transformation). Mann–Whitney test was used for group comparison of skewed variables. Categorical variables were compared with Chi-square test or Fischer’s exact test, in the case of few observed cases (*n *< 5). All statistical significance was set as alpha significance 0.05.

## Results

We enrolled 743 pregnant women from 25 MHC centers (18% of all MHCs asked). The recruitment of the 25 MHC centers (Table 1) followed the intended plan, with one exception: the region with the highest population nativity (H1 region) contributed two out of the intended six MHC centers. The remaining four MHC centers were recruited from the H2 region, i.e. the region with the second highest population nativity. Subject characteristics are presented in Table 2. Iodine-containing supplements (≥ 150 µg iodine/day) were used by 34.8% of the women.

**Table 1:** Recruitment at Maternal Health Care (MHC) centers in Sweden based on statistically determined number of MHC centers in each H region

Region	H1	H2	H3	H4	H5	H6	Total
Maternal Health Care centers	Sundbyberg Vallentuna	Malmö Majorna Stenungsund Lund Kungälv Angered Backa Linnestaden Brämaregårdens	Norrköping Karlshamn Helsingborg Halmstad Umeå Borås Skövde Falun Gävle	Vetlanda Varberg Avesta	Östersund	Strömsund	
Intended recruitment (*n*)	180	150	270	90	30	30	756
Actual recruitment (*n*)	60	271	263	89	30	30	743

The six H regions represent geographical areas in Sweden with different population nativity (H1 with the highest nativity trough H6 with the lowest nativity)

**Table 2:** General characteristics of the study participants in a nationally representative sample of pregnant women in Sweden

		No. of participants evaluated
Median gestational age, weeks (IQR)		
All participants	23 (9, 38)	741
First trimester	10 (9, 11)	259
Second trimester	24 (18, 25)	235
Third trimester	34 (30, 36)	247
Multiple pregnancy, *n* (%)		
No	533 (72)	743
Yes	23 (3)	
Unknown	187 (25)	
Median BMI, kg/m^2^ (IQR)		
First trimester	23.8 (21.9, 26.7)	251
Second trimester	25.3 (23.3, 28.7)	231
Third trimester	28.4 (26.3, 31.6)	240
Smoker, *n* (%)		
No	719 (97)	
Yes	18 (2)	743
Unknown	6 (1)	
Dietary supplement use, *n* (%)		
None	376 (51)	742
Unknown iodine content	43 (6)	
< 75 μg iodine/day	31 (4)	
75–100 μg iodine/day	34 (5)	
101–149 μg iodine/day	0 (0)	
≥ 150 μg iodine/day	258 (35)	

*BMI* body mass index, *IQR* interquartile range

**Fig. 1:**
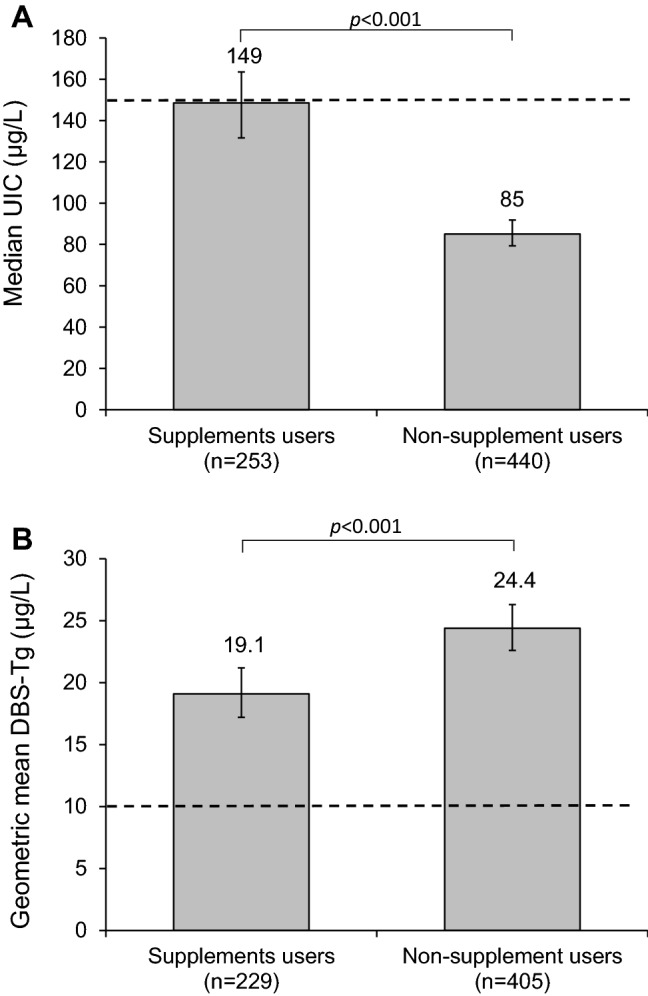
Median (boostrapped 95% CI) UIC (**a**) and geometric mean (95% CI) DBS-Tg (**b**) in pregnant women who consume a daily supplement of ≥ 150 μg iodine (supplement users) *vs* those who consume no supplements or supplements containing < 150 μg iodine (non-supplement users). Dotted line represents the lower recommended median UIC during pregnancy (**a**) and the Tg level expected in an iodine-sufficient population (**b**) [18, 28]. Mann–Whitney *U* test and unpaired *t* test were used to test differences between the groups. *CI* confidence interval, *DBS-Tg* dried blood spot thyroglobulin concentration, *UIC* urinary iodine concentration

The overall median UIC (IQR) was 101 µg/L (61, 182; *n *= 737) with bootstrapped 95% confidence interval (CI) (95, 108), without difference across trimesters (*p *= 0.381, Table 3). The UIC:U-creatinine ratio (IQR) was 0.11 (0.07, 0.19; *n *= 737), with no difference across trimesters (p = 0.153, Table 3). The median UIC (IQR) was 149 µg/L (88, 253; *n *= 253) in supplement users (using supplements containing ≥ 150 µg iodine/day) and 85 µg/L (51,134; *n *= 440) in non-supplement users (using no supplements or supplements containing < 150 µg iodine/day) (*p *< 0.001). The bootstrapped 95% CI of median UIC was (132, 164) in supplement users and (79, 92) in non-supplement users (Fig. 1a). Levothyroxine was used by 8.9% (*n *= 66) of the women, being equally distributed among supplement and non-supplement users (*p *= 0.206). The median UIC after excluding those on levothyroxine treatment did not differ compared to the median UIC in the complete study group (*p *= 0.942, data not shown).

**Table 3:** Iodine status, thyroid function tests and thyroid morbidity in a nationally representative sample of pregnant women in Sweden

		No. of participants evaluated	*p* value
Median UIC μg/L (IQR)			
All participants	101 (61, 182)	737	0.381
First trimester	105 (69, 177)	254	
Second trimester	97 (60, 181)	235	
Third trimester	100 (60, 183)	246	
Median U-creatinine μg/L (IQR)			
All participants	1018 (571, 1528)	737	–^a^
First trimester	1131 (665, 1696)	254	
Second trimester	860 (531, 1357)	235	
Third trimester	972 (531, 1471)	246	
Median UIC:creatinine ratio (IQR)			
All participants	0.11 (0.07, 0.19)	737	0.153
First trimester	0.10 (0.06, 0.18)	254	
Second trimester	0.11 (0.08, 0.21)	235	
Third trimester	0.11 (0.07, 0.19)	246	
Geometric mean DBS-Tg, μg/L (95% CI)			
All participants	22.1 (20.8, 23.5)	675	0.579
First trimester	22.8 (19.6, 24.3)	223	
Second trimester	22.1 (18.9, 23.5)	209	
Third trimester	23.3 (21.0, 25.9)	230	
Median DBS-TSH, mU/L (IQR)			
All participants	0.7 (0.5, 1.0)	672	–^a^
First trimester	0.7 (0.5, 0.9)	234	
Second trimester	0.7 (0.5, 1.0)	208	
Third trimester	0.8 (0.5, 1.0)	228	
Median DBS-tT4, nmol/L (IQR)			
All participants	124 (90, 155)	672	–^a^
First trimester	120 (91, 152)	234	
Second trimester	127 (91, 158)	208	
Third trimester	124 (84, 157)	228	
Positive S-TPOabs, *n* (%)			
All participants	39 (11.6)	334	
First trimester	19 (16.6)	114	
Second trimester	11 (10.6)	104	0.100
Third trimester	9 (7.8)	116	
Clinical thyroid disease, *n* (%)	0	669	–
Subclinical hyperthyroidism, *n* (%)	0	669	–
Subclinical hypothyroidism, *n* (%)			
All participants	2 (0.3)	669	0.333
First trimester	2 (0.3)	233	
Second trimester	0	208	
Third trimester	0	228	
Isolated hypothyroxinemia, *n* (%)			
All participants	166 (24.8)	669	0.002^b^
First trimester	41 (17.6)	233	
Second trimester	53 (25.5.)	208	
Third trimester	72 (31.6)	228	
Isolated hyperthyroxinemia, *n* (%)			
All participants	32 (4.8)	669	0.033^c^
First trimester	18 (7.7)	233	
Second trimester	7 (3.4)	208	
Third trimester	7 (3.1)	228	

Subjects treated with levothyroxine (*n *= 66) are excluded

*CI* confidence interval, *DBS* dried blood spot, *IQR* interquartile range, *S-TPOabs* serum thyreoperoxidase antibodies, *Tg* thyroglobulin, *TSH* thyroid-stimulating hormone, *tT4* total thyroxine, *UIC* urinary iodine concentration, *U-creatinine* urinary creatine concentration

^a^Analysis between trimesters not conducted, due to lack of clinical relevance (regarding U-creatinine) or due to different reference range (regarding DBS-TSH and DBS-tT4)

^b^Analysis between trimesters for isolated hypothyroxeinemia: First-Second trimester, *p *= 0.044; Second-Third trimester; *p *= 0.160, First-Third trimester, *p *< 0.001

^c^Analysis between trimesters for isolated hyperthyroxinemia: First-Second trimester, *p *= 0.048; Second-Third trimester, *p *= 0.861; First-Third trimester, *p *= 0.027

The overall geometric mean DBS-Tg (95% CI), after exclusion of subjects on levothyroxine (*n* = 66) was 22.1 μg/L (20.8, 23.5; *n *= 675), with no difference across trimesters (*p *= 0.579, Table 3). The geometric mean DBS-Tg (95% CI) was 19.1 µg/L (17.2, 21.3, *n *= 229) in supplement users and 24.4 µg/L (22.6, 26.3, *n *= 405) in non-supplement users (*p *< 0.001) (Fig. 1b). The prevalence of elevated DBS-Tg was 19%: 13.1% among supplement users and 22.5% of among non-supplement users (*p *= 0.004). After exclusion of subjects with S-TPOab positivity (*n* = 45), the geometric mean DBS-Tg was still lower (*p *= 0.002) in supplement users than in non-supplement users (data not shown).The concentration of thyroid function parameters (DBS-TSH, DBS-tT4, and S-TPOabs) and the prevalence of subclinical and clinical thyroid disorders after exclusion of subjects treated with levothyroxine (*n *= 66) are presented in Table 3. The prevalence of thyroid dysfunction was low overall, except for isolated hypothyroxinemia, present in 24.8% of pregnant women (Table 3). When stratified for trimesters, the frequency of isolated hypothyroxenimia was higher and isolated hyperthyroxenimia lower in third trimester than in the first trimester. Comparison between first and second trimester gave just marginal significances of the same direction, whereas second and third trimesters presented no differences (Table 3). Clinical thyroid disease and subclinical hyperthyroidism were not observed. There was no difference between supplement users and non-supplement users for: prevalence of levothyroxine medication (*p *= 0.206, *n *= 699), DBS-TSH (*p *= 0.633, *n *= 633), DBS-tT4 (*p *= 0.328, *n *= 633), and S-TPOab positivity (*p *= 0.107, *n *= 318). The two groups did not differ for any of the three entities of thyroid morbidity observed: subclinical hypothyroidism (*p *= 1.0, *n *= 631), isolated hypothyroxinemia (*p *= 0.633, *n *= 631), and isolated hyperthyroxinemia (*p *= 0.449, *n *= 631). After exclusion of S-TPOab positive subjects (*n* = 45), no difference was observed between supplement users and non-supplement users for DBS-TSH and DBS-tT4 (data not shown).

## Discussion

This is the first national study assessing iodine status and thyroid function of pregnant women in Sweden. The median UIC of 101 μg/L in the study population is far below the recommended optimal range for pregnant women of 150–249 μg/L [10] and the results suggest mild iodine deficiency.

Our results are consistent with previous data. Local and regional studies in pregnant women in Sweden, conducted more than 10 years ago [19, 30], report low UIC and elevated Tg concentration, indicating that iodine intake in pregnant women has been insufficient over a prolonged period. However, the overall iodine intake in school-aged children is adequate. A national study in children aged 6–12 years conducted by our group in 2007 found a median UIC of 125 μg/L [17], which is within the recommended range of 100–299 μg/L [10, 18], and goiter was not observed [16].

Iodine deficiency during pregnancy is frequently observed despite iodine sufficiency in school-aged children, e.g. in Norway, Denmark, Switzerland, USA [14, 31–33]. A review of studies conducted simultaneously in school-aged children and pregnant women in countries where salt is the main dietary source of iodine, showed that median UIC in pregnant women typically meets the recommended 150 μg/L in populations where the median UIC in school-aged children exceeds 180 μg/L [34]. However, in countries with voluntary iodization, the coverage by iodized salt may be incomplete. Although the data are limited (personal communication with the salt industry, 2017), most of the household salt in Sweden appears to be iodized, whereas processed food and food served in restaurants, an important salt source for working women in Sweden, seems to have lower coverage by iodized salt. Therefore, despite voluntary iodization at 50 mg/kg salt [14, 15], above the recommended 20–40 mg/kg [10], iodine intake in Sweden is inadequate in pregnant women, probably due to poor coverage by iodized salt. A recent study conducted in populations with mandatory legislation and high coverage by iodized salt [35] demonstrated that a well implemented salt iodization program ensures adequate dietary intake in all population groups. National recommendations in Sweden to promote iodized salt by the food industry are warranted as well as consideration of applying this on a mandatory basis.

One-third of the women in our study consumed iodine-containing prenatal dietary supplements containing ≥ 150 μg iodine/day and the median UIC in iodine supplement users suggested adequate iodine intake. The additional supplemental iodine may have a positive impact on the thyroid gland, as suggested by the lower DBS-Tg in this group compared to non-supplement users. However, the DBS-Tg concentration in supplement users was still higher than expected for an iodine-sufficient population (~ 10 μg/L) [25]. It is possible that women in Sweden have inadequate iodine intake before conception and iodine supplementation is not sufficient to normalize DBS-Tg during pregnancy. This is supported by the elevated DBS-Tg observed in the first trimester. It is also possible that other nutrient deficiencies beyond iodine (e.g. selenium, iron [36, 37]) interact with thyroid metabolism. Finally, thyroid size, reflected by DBS-Tg, may be larger in the Swedish population, as shown for school-aged children when compared with an age-matched reference international population [16].

The overall prevalence of isolated hypothyroxinemia was 25% and did not differ between supplement users and non-users, but was higher in third trimester (32%) compared with first trimester (18%). The high prevalence of isolated hypothyroxinemia in our population contrasts with a local Swedish study [38], where only 2.8% of pregnant women presented isolated hypothyroxinemia, which was though measured in serum samples at median pregnancy week 18. The clinical importance of isolated hypothyroxinemia is debated; some studies indicate an association with impaired neurocognitive development of offspring [39], whereas others do not [40, 41]. The use of tT4 concentration, as a proxy for free T4, may induce bias when estimating the prevalence of hypothyroxinemia. Increased estradiol production during pregnancy leads to elevation of thyroxine-binding globulin, resulting in higher tT4 despite stable or lower free T4 during pregnancy. To compensate for this, an elevation of the upper limit of the reference range throughout pregnancy is recommended [26] and was applied in our study, but the precision of the lower limit of tT4 is uncertain. Furthermore, no reference range has specifically been defined for DBS-T4 and DBS-TSH during pregnancy, possibly contributing to misclassification. Uncertainty also prevails in terms of the free T4 reference range during pregnancy, free T4 being associated with tT4. In one study [42], free T4 decreased from first to second trimester despite iodine supplementation and TSH remained stable, suggesting the decrease in free T4 during pregnancy may be a physiological observation. The same trend during pregnancy was observed in our study population regarding isolated hypothyroxinemia; the opposite trend, though in lower prevalence, was noticed in isolated hyperthyroxenemia, which was more frequent in first trimester (8%) than in third trimester (3%), highlighting the need of proper reference intervals deriving from a thyroid healthy, iodine sufficient population of pregnant women.

The observed mild iodine deficiency in this study raises the question of national recommendation in Sweden for iodine supplementation of 150 µg/day during pregnancy, as recommended in several other countries [26, 43] and as supported by World Health Organization when the coverage of iodized salt is incomplete [10]. Several observational studies suggest an association between mild iodine deficiency during fetal life and poor educational level, motor skills, or verbal abilities in children 3–12 years of age [5, 6, 44, 45]. However, this was not confirmed in randomized controlled trials of iodine supplementation of pregnant women with mild iodine deficiency [8, 42], possibly due to lower than expected prevalence of iodine deficiency in the population studied in one trial [8] or due to low sample size in the other trial [42]. At the same time, other observational studies have indicated the opposite, i.e. iodine supplementation during fetal life was associated with higher risk of behavioral problems or poorer mental and psychomotor development in children [45–47]. It has been proposed that a sudden increase of iodine intake blocks fetal thyroid function, leading to hypothyroidism and goiter, especially in the case of preconceptional iodine deficiency [48]. Women with preconceptional iodine deficiency may handle the restricted available iodine more effectively during pregnancy [49]. Taken together, the data supporting iodine supplementation during pregnancy in mild ID are inconclusive. A randomized clinical trial of prenatal iodine supplementation is currently ongoing in Sweden and the results will provide valuable data on the consequences of mild iodine deficiency on fetal development [50].

We recognize the low response rate of the MHC centers (18% of all asked) as a weakness of the study, but the reason given for declining participation was the high workload of midwives, which is unlikely to be associated with the outcome parameters. Although only two of the intended six MHC centers were included from the H1 region, the remaining four MHC centers were recruited from the region (H2) with the closest population density. The applied stratified cluster sampling methodology still ensured a representative sample of the Swedish pregnant population. Data on iron and selen status or other endocrine disruptors were not collected; we plan, however, to investigate the possible interaction between iodine, iron and selen within the framework of another study (ClinicalTrials.gov identifier: NCT02378246). We did not study the locality either, as sample size for each area was small and we followed the WHO recommendations for monitoring of iodization program by clinic-based cross-sectional surveys (10). Besides, a comparison between the different areas in the country was beyond the scope of this study, as we aimed to set the ground for recommendations to pregnant women nationally.

In conclusion, adequate iodine intake in school-aged children does not guarantee adequate intake in risk populations, even in a country with a high level of iodine fortification of salt. Pregnant women in Sweden have inadequate iodine nutrition and only 35% consume a daily supplement with iodine ≥ 150 μg. Women not taking iodine-containing supplements or supplements containing < 150 μg/day are exposed to mild iodine deficiency. The low iodine intake increases the risk of elevated thyroid activity to maintain euthyroidism. The long-standing national policy of universal salt iodization on a voluntary basis is not operating as intended and actions to improve its coverage must be urgently undertaken. We suggest that Swedish authorities encourage the food industry to use iodized salt instead of non-iodized salt and we urge regular monitoring of iodine status of the general Swedish population and of groups at particular risk of inadequate iodine intake, as a part of the national nutrition policy, supported by the National Food Agency. A randomized controlled trial in a population with mild iodine deficiency is highly warranted [50] to provide authorities with guidance on whether iodine supplementation during pregnancy should be recommended or not. If the coverage of iodized salt is not improved and mild iodine deficiency infers mental consequences, mandatory iodization of table salt may be considered.
